# Dynamic functional network connectivity discriminates mild traumatic brain injury through machine learning

**DOI:** 10.1016/j.nicl.2018.03.017

**Published:** 2018-03-15

**Authors:** Victor M. Vergara, Andrew R. Mayer, Kent A. Kiehl, Vince D. Calhoun

**Affiliations:** a The Mind Research Network and Lovelace Biomedical and Environmental Research Institute, 1101 Yale Blvd. NE, Albuquerque, NM 87106, United States; b Dept of ECE, University of New Mexico, Albuquerque, NM 87131, United States; c Department of Psychology, University of New Mexico, Albuquerque, NM 87131, United States; d Department of Neurology, University of New Mexico School of Medicine, Albuquerque, NM 87131, United States; e Department of Psychiatry and Behavioral Sciences, University of New Mexico School of Medicine, Albuquerque, NM 87131, United States

**Keywords:** Traumatic brain injury, Magnetic resonance imaging, Dynamic functional network connectivity

## Abstract

Mild traumatic brain injury (mTBI) can result in symptoms that affect a person's cognitive and social abilities. Improvements in diagnostic methodologies are necessary given that current clinical techniques have limited accuracy and are solely based on self-reports. Recently, resting state functional network connectivity (FNC) has shown potential as an important imaging modality for the development of mTBI biomarkers. The present work explores the use of dynamic functional network connectivity (dFNC) for mTBI detection. Forty eight mTBI patients (24 males) and age-gender matched healthy controls were recruited. We identified a set of dFNC states and looked at the possibility of using each state to classify subjects in mTBI patients and healthy controls. A linear support vector machine was used for classification and validated using leave-one-out cross validation. One of the dFNC states achieved a high classification performance of 92% using the area under the curve method. A series of *t*-test analysis revealed significant dFNC increases between cerebellum and sensorimotor networks. This significant increase was detected in the same dFNC state useful for classification. Results suggest that dFNC can be used to identify optimal dFNC states for classification excluding those that does not contain useful features.

## Highlights

• Dynamic functional connectivity and support vector machine classified traumatic brain injury patients and healthy controls.
• Out of 4 dynamic brain states, we identified 1 state useful for classification.
• Classification performance of the dynamic state of interest achieved a performance of 92% area under the curve method.

## Graphical abstract

**Figure:**
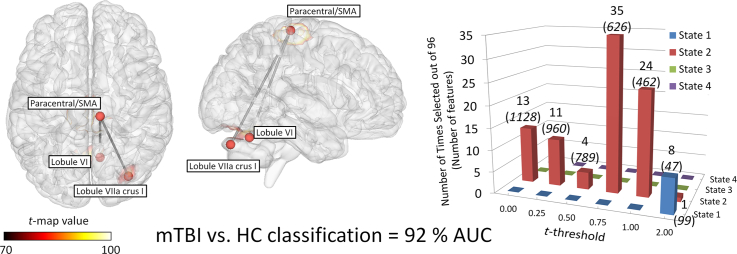

## 1 Introduction

Mild traumatic brain injury (mTBI) symptoms can affect a person's cognitive and social faculties. Although symptoms might resolve within the first three months after the trauma, some patients continue having related deficits that may persistent through their life (Levin and Diaz-Arrastia, 2015). Mild TBI complications include chronic headaches, dizziness, vertigo, difficulty concentrating, depression, irritability, and impulsiveness (DeKosky et al., 2010). In spite of the important influence of mTBI in patients, misdiagnosis is common (Kristman et al., 2014). According to the World Health Organization and the National Academy of Neurology existing mTBI diagnosis methods provide limited evidence of their validity (Borg et al., 2004; Ruff et al., 2009). Alternative diagnosis methods, some based on magnetic resonance imaging, are motivating current research efforts that promise better detection of mTBI (Huisman et al., 2004; Lipton et al., 2012; Narayana et al., 2015; Vergara et al., 2017). Further refinement of these new technologies is important to achieve performances applicable in clinical settings.

An important observation in mTBI is the existence of microstructural axonal injuries affecting white matter in areas including genu and splenium of the corpus callosum, the corona radiata, and the internal capsule (Arenth et al., 2014; Holli et al., 2010; Huisman et al., 2004; Ling et al., 2012). Consequently, white matter injuries can also affect the connectivity among grey matter areas, translating into dysfunctional connectivity (Hillary et al., 2014; Mayer et al., 2015a; Sharp et al., 2014). Dysfunctions have been found in mTBI patients in the default mode network (DMN) (Mayer et al., 2011; Sours et al., 2013; Zhou et al., 2012). A set of weaker functional connectivity observations have been found between several pairs of brain areas including DMN-basal ganglia, attention-sensorimotor, attention-frontal, and within the sensorimotor networks (Vakhtin et al., 2013). The thalamus, as an important area of information relay, has been involved in abnormal functional connectivity including thalamo-thalamo, thalamo-frontal, and thalamo-temporal circuits (Tang et al., 2011; Zhou et al., 2014). Slobounov et al. found connectivity disruptions among primary visual, hippocampal, and dorsolateral prefrontal cortexes (Slobounov et al., 2011). In contrast, increased connectivity has been observed in the cerebellum (Nathan et al., 2015). These findings show a picture of important dysfunctions distributed through cerebrum and cerebellum that might be used as important features for the detection of mTBI. Since abnormal connectivity in mTBI might not be specific to a brain area, previously observed dysfunctions might need to be treated as an ensemble rather than considering separate parts of the brain.

Our group has found evidence that including a large ensemble of functional connectivity differences in one analysis can be promising for the development of mTBI biomarkers (Vergara et al., 2017). Additionally, the same study found that changes in static functional network connectivity (sFNC) were statistically significant among cerebellum, precuneus, temporal and supplementary motor area (SMA) in mTBI patients during the semi-acute stage. However, high accuracy in discriminating mTBI was only achieved after including a large set of functional connectivity assessments with relatively weak group differences. The sFNC study was based on the Pearson correlation coefficient, one of the most simple and widely utilized measures of functional connectivity (Allen et al., 2011). Temporal correlation between two areas of brain is measured in relatively long time periods of approximately 5 min or more. One limitation of this method is that sFNC represents a summary of the connectivity over the considered time interval, but excludes temporal dynamics from the analysis (Hutchison et al., 2013). In spite of assuming static connectivity, sFNC achieved high classification performance, 84% area under the curve (AUC), using a group of 96 samples (48 mTBI and 48 matched healthy controls).

The study of temporal changes of functional connectivity among spatially separated areas of the brain has been hypothesized to provide important information for the understanding of neurodegenerative diseases that might not be accessible through static connectivity (Sakoğlu et al., 2010). The dynamic connectivity analysis of resting state data has revealed the existence of short lived coactivation patterns occurring in temporal succession (Allen et al., 2014). While sFNC could be seen as an aggregation of occurring dynamic changes, a mapping from observed dynamic coactivations to the pattern observed in sFNC is not easy to define. One of the critical reasons for this difficulty is that methods for the estimation of dynamic connectivity may include non-linear operations such as clustering (Hutchison et al., 2013). Thus, results from both techniques might be different and must be compared to determine differences in their performance as biomarkers.

Here we have reanalyzed the resting state data from our previous study (Vergara et al., 2017) using a dynamic functional network connectivity (dFNC) approach (Allen et al., 2014; Hutchison et al., 2013; Yaesoubi et al., 2015). Whole brain connectivity was separated into dynamic states (a finite set of coactivation patterns), each carrying different connectivity characteristics. We hypothesize that some states might be better biomarker candidates than others. The final objective of the current analysis is to identify dFNC states strongly affected by mTBI and utilize these effects as biomarkers. In order to discriminate mTBI subjects from healthy controls we applied a support vector machine and cross-validated the results.

## 2 Material and methods

### 2.1 Subjects

The sample cohort has been utilized previously to study different sets of brain data modalities (Ling et al., 2012; Mayer et al., 2015b) including analyses of static and dynamic connectivity (Mayer et al., 2015a; Vergara et al., 2017). Data from one hundred subjects were available. Four subjects were excluded due to high movement variance for a total of 96 subjects. In this cohort, a total of 48 mTBI patients and 48 healthy controls (HC) were matched by sex, age (up to 3 years) and years of education with no significant group difference (*p* > 0.05). The Wechsler Test of Adult Reading (WTAR) was included as clinical variable with significant differences between HC and mTBI subjects. Table 1 displays more complete information about these demographics.

**Table 1:** Demographics per dFNC States. The * symbol indicates significant difference. Differences of sex were evaluated using Fisher's exact test (Routledge, 2005).

	HC mean	HC std	mTBI mean	mTBI std	*t*-Value (mTBI-HC)	*p*-Value
All subjects						
Sex	Males = 23	Females = 25	Males = 23	Females = 25		1.00
Age	27.40	8.96	27.79	9.18	0.21	0.83
Edu	13.92	2.13	13.13	2.25	−1.77	0.08
WTAR	55.50	7.40	50.10	8.74	−3.30	*0.0014

State 1						
Sex	Males = 21	Females = 22	Males = 16	Females = 21		0.66
Age	27.84	9.31	28.03	9.23	0.09	0.93
Edu	14.05	1.96	13.14	2.34	−1.90	0.06
WTAR	56.13	7.31	50.50	8.23	−3.26	*0.0016

State 2						
Sex	Males = 22	Females = 21	Males = 23	Females = 23		1.00
Age	27.77	9.37	27.98	9.28	0.11	0.92
Edu	13.79	2.05	13.17	2.24	−1.35	0.18
WTAR	55.56	7.28	50.24	8.83	−3.09	*0.0027

State 3						
Sex	Males = 10	Females = 10	Males = 15	Females = 11		0.77
Age	28.40	10.94	27.04	8.35	−0.48	0.63
Edu	13.35	2.41	12.65	2.35	−0.99	0.33
WTAR	57.70	6.51	47.65	9.76	−3.97	*0.0003

State 4						
Sex	Males = 12	Females = 14	Males = 13	Females = 11		0.78
Age	27.73	9.66	27.92	10.64	0.06	0.95
Edu	13.92	2.17	12.58	2.39	−2.08	*0.04
WTAR	55.50	7.39	48.95	8.33	−2.94	*0.0050

The mTBI patients went through clinical (mean day post-injury = 13.9 ± 4.9 days) and brain imaging (mean day post-injury = 14.0 ± 5.3 days) evaluations within 21 days of injury. The maximum time between clinical and imaging sessions was 6 days (mean interval between sessions = 1.3 ± 1.6 for the mTBI group).Patients were recruited from local emergency rooms with inclusion criteria based on the American Congress of Rehabilitation Medicine. Subjects classified as mTBI had a Glasgow Coma Scale (Teasdale and Jennett, 1974) between 13 and 15, a maximum of 30 min loss of consciousness (if present), and a maximum of 24 h post-traumatic amnesia (if present). Subjects were excluded if there was a history of neurological disease, major psychiatric disturbance, and additional closed head injuries with >5 min of lost consciousness, additional closed head injury within the past year, learning disorder, ADHD, or a recent history of substance abuse/dependence including alcohol. All participants provided informed consent according to the Declaration of Helsinki and the institutional guidelines at the University of New Mexico.

### 2.2 Imaging protocol

All images were collected on a 3 Tesla Siemens Trio scanner. Each participant completed a 5-minute resting state run using a single-shot, gradient-echo echo planar pulse sequence (TR = 2000 ms; TE = 29 ms; flip angle = 75°; FOV = 240 mm; matrix size = 64 × 64). Foam padding and paper tape were used to restrict motion within the scanner. Thirty-three contiguous, axial 4.55-mm thick slices were selected to provide whole-brain coverage (voxel size: 3.75 × 3.75 × 4.55 mm) during the resting state scan. The first five images were eliminated to account for *T*_1_ equilibrium effects; 145 images were selected for further analysis. Presentation software (Neurobehavioral Systems) was used for stimulus presentation and synchronization of stimuli with the MRI scanners. Subjects were instructed to passively stare at a foveally presented fixation cross (visual angle = 1.02°) for approximately 5 min and to keep head movement to a minimum.

### 2.3 fMRI pre-processing

Preprocessing and other analyses were similar to our previous publication and are therefore only briefly presented here (Vergara et al., 2017). Data were pre-processed using statistical parametric mapping (Friston, 2003) (SPM v5: http://www.fil.ion.ucl.ac.uk/spm) including slice-timing correction, realignment, co-registration, and spatial normalization and then transformed to the Montreal Neurological Institute standard space. For despiking we utilized the command 3dDespike from the software Analysis of Functional NeuroImages (AFNI v17.1.03). The time courses were also orthogonalized with respect to the following: i) linear, quadratic, and cubic trends; ii) the six realignment parameters; and iii) realignment parameters derivatives. A full width at half maximum Gaussian kernel of 6 mm was then used for smoothing. Data from all subjects were subject to a gICA (Calhoun et al., 2001; Calhoun and Adali, 2012) using the GIFT software (GIFT v4: http://mialab.mrn.org/software/gift/) to obtain a set of functionally independent components. Each independent component delimits a network of brain regions that may be either adjacent or spatially separated. Given the resting state nature of the fMRI data the components are designated as resting state networks (RSNs) (Calhoun et al., 2008). The data of each RSN consists of a spatial map of involved brain regions and one associated time course characterizing temporal behavior. The gICA technique find a set of independent RSNs, but it is not designed to estimate how many RSNs should be considered. RSNs should be independent of each other as well as replicable, but the number of RSNs requested from gICA may change these characteristics. The software package ICASSO (Himberg and Hyvarinen, 2003; Himberg et al., 2004), currently integrated in GIFT, was utilized to assess the quality of the RSNs. In addition, ICASSO runs gICA multiple times (10 times in our case) to pick the centroid result, thus mitigating the issue of multiple answers for each random starting point. ICASSO supply an R-index as a measure of compactness and separation among RSNs, with lower magnitude indicating better features. In addition, ICASSO calculate a quality index per RSN in the range [0 1] with higher values indicating better components. The number of components was determined to be 70 using the version of ICASSO implemented in the GIFT package (Himberg et al., 2004; Ma et al., 2011) such that the overall R-index is close to the minimum and the quality index of any given RSN is above 0.7. The minimum R-index is an indicator of the best number of components as suggested by Himberg et al. (Himberg et al., 2004). However, the same author recommends high RSN quality indexes (Himberg and Hyvarinen, 2003). The procedure consisted of running gICA with different numbers of components and selecting the lowest R- and the highest RSN quality indexes. This setup provided good consistency trade-off between RSN quality and number of components considering. A fifth-order Butterworth band-pass filter [0.01 0.15] Hz was applied to the time courses of each component as it has been previously proposed in the literature (Allen et al., 2014; Allen et al., 2011; Miller et al., 2016).

Artifactual components were detected and discarded based on their frequency power spectrum content following the procedure in (Allen et al., 2011). RSNs were also manually inspected and classified into broader categories or discarded if their main activation occurs in areas of white matter or cerebrospinal fluid. A subset of 48 RSNs was selected. Spatial maps and MNI coordinates for the selected RSNs are displayed in Supplementary Fig. 1. These RSNs were organized in nine groups: subcortical (SBC), cerebellum (CER), auditory (AUD), sensorimotor (SEN), visual (VIS), salience (SAL), default mode (DMN), executive control (ECN) and language (LAN).

A dFNC analysis was performed using the dynamic FNC Toolbox (dFNC v1.0a) available in the GIFT package. The dFNC sliding window size was set to 15 TRs (30 s) of a rectangular window convolved with a Gaussian (*σ* = 3 TRs). To find dFNC states we utilized k-means clustering. The number of clusters was selected by running k-means for values of k from 2 to 9 and using the elbow criteria. The clustering index results for different k-values used to determine the number of states can be found in Supplementary Fig. 2. The number of states was determined to be 4. For each subject, the k-means algorithm provided a match between cluster membership and moments in time. We separated the time intervals belonging to each state for each subject. A representative dFNC matrix for each state was calculated on each subject by averaging dFNC matrices of the same state. Information on temporal state membership by subject can be found in Supplementary Fig. 3. Subsets of subjects were separated for each state based on the temporal membership and used for further analysis. Not all subjects visited all states, thus the total number of subjects on each state varies and is less than the 96 available subjects. Distribution of subjects among dFNC states, as well as state-wise demographic information, is displayed in Table 1. Years of education in State 4 exhibited significant group difference. All dFNC states had significant WTAR differences between HC and mTBI.

### 2.4 Diagnosis performance

We seek dFNC differences between mTBI and HC on each state separately. A two sample *t*-test was used for each dFNC, i.e. each element on the dFNC matrix, within a given state. As previously explained, each subject has a representative dFNC matrix for each state visited. Since not all subjects visited all states, we utilized the available subject representative dFNC matrices to perform 1128 (since there are 48 RSNs there are 48 ∗ 47/2 = 1128 dFNC connectivity values) *t*-tests in each state. The *p*-values were corrected using the false discovery rate (FDR) and significant group differences assessed at *p* < 0.05. The set of 1128 *t*-values in each state were saved for later use. A linear support vector machine (LSVM) was utilized to classify subjects into mTBIs and HCs on each of the dFNC states. The least square method was used to solve the LSVM with a soft margin parameters C = 0.1. Classification accuracy was assessed using the area under the curve (AUC) measure.

Since not all subjects visited all states, only the subset of subjects within a given state was used for classification. The set of subjects for each state was the same as that used for the *t*-test group analysis. The first analysis applied an LSVM for each state. We utilized the whole set of 1128 correlations as classification features. Since the total number of samples is low (96 samples), and state subsets have fewer subjects, we used a leave one out cross validation (LOOCV) to measure one AUC per state. Each iteration consisted of separating the 1128 dFNC values from one subject to be used as testing data and using the remaining data to train a LSVM. Classification was performed by feeding the testing data to the trained LSVM. This procedure was applied to all subsets of subjects corresponding to each state. These results will allow us to determine the LSVM performance expected from each state.

A more complete analysis was implemented performing feature and state selection on the training sample of each of the LOOCV iterations. After leaving one testing sample out, an optimization algorithm was applied to the set of training samples in a secondary nested LOOCV loop, as it has been previously utilized (Hahn et al., 2015; Vergara et al., 2017; Whelan et al., 2014), to each combination of feature set and state. If the left out sample was not found in a given state then this state was not included in the nested optimization loop. Feature selection was driven by the *t*-values previously obtained. In this case *t*-values larger than a *t*-threshold were chosen. Six different *t*-thresholds were tested in the training set resulting in different number of features for each LOOCV loop. The combination of dFNC state and *t*-threshold with the largest AUC of all nested LOOCV loops was then selected to classify the left out sample. This way, a final classification performance was obtained along with the parameter stability. This stability is based on how consistent a given parameter (*t*-threshold and dFNC state) was chosen in the LOOCV.

## 3 Results

**Fig. 1:**
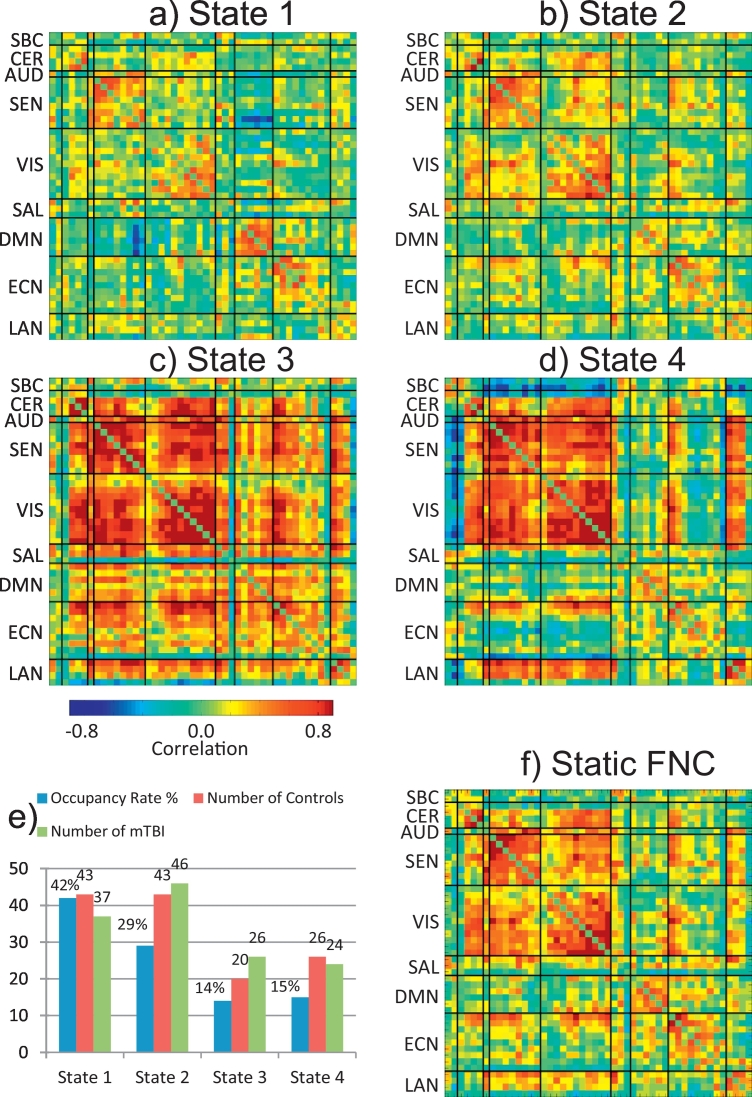
Centroids obtained for the four dFNC states (a–e). The figure includes the mean static FNC matrix (f), the occupancy rates and the number of subjects in each state (e). State 1 had the largest occupancy rate and State 3 the smallest. The number of mTBI and controls on each state are similar.

Centroids for each of the four dFNC states are displayed in Fig. 1(a–d). The figure includes the occupancy rates (Fig. 1e) expressed in percentage with State 1 having the largest occupancy. Fig. 1f includes the sFNC matrix for visual comparison with the dFNC states. For each subject, a representative dFNC state was calculated by averaging all dFNC windows belonging to the same state. However, not all dFNC states were detected in all subjects. The number of subjects entering each state was counted and included in Fig. 1e. In this case most of the subjects (89 out of 96) visited State 2 and State 1 (80 out of 96), which are also the states with largest occupancy rates. A total of 46 subjects visited State 3 and 50 visited State 4. The number of controls and mTBI subjects are similar in each state indicating that although having a reduced number of subjects, both groups are equally represented.

**Fig. 2:**
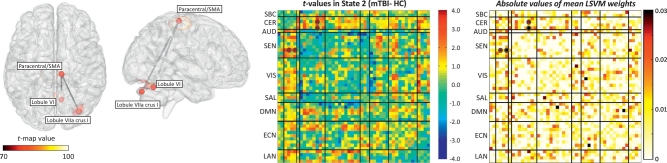
Connectivity difference results for State 2 evaluated using two-sample *t*-tests. Significance was corrected using false discovery rate (FDR) over the total of 1128 (48 ∗ 47/2) dFNC values on that state. Each displayed vertex represents a dFNC increment in mTBI compared to HC. No *t*-test survived FDR correction in any of the other three states. The matrix with all *t*-values for State 2 is displayed and the circles indicate the significant (FDR corrected) *t*-tests. The mean SVM weight vector is also displayed where similarities with the *t*-value matrix can be observed.

Two sample *t*-tests were performed on each of the 1128 mean dFNC correlations (48 ∗ 47/2) from each of the 4 states. Mean dFNC matrices for each group (HC and mTBI) along with *t*-value matrices are included in Supplementary Fig. 4. The *p*-values were corrected using false discovery rate (FDR). Only two group differences were found in State 2 where mTBI exhibited stronger dFNC compared to controls. The *t*-test results are illustrated in Fig. 2 displaying the location of RSN peaks. In addition, a regression model with sex, age, education and WTAR as independent variables was performed. The sex regressor was significant in both cases and education only for the RSN pair R Lob.VIIa Crus I – SMA/Paracentral. More information is provided in Table 2 where MNI coordinates for peaks, *t*- and *p*- values are provided. Although there was difference between HC and mTBI, the clinical variable WTAR had no significant result in the regression model. This might be linked to the lack of cognitive or language brain areas involved in the regression test. These two group differences were found in similar areas previously reported in static FNC (Vergara et al., 2017), but in these dFNC results the effect size is larger. Finding these outcomes is important because it points to the source of the effect as a strong dysfunction in State 2. Although being strongly related, there is no guarantee that static and dynamic connectivity will be sensible to the same dysfunctions. One can consider dFNC to be more specific than static FNC since it is unpacking temporal features otherwise averaged in static FNC measures. Similarly, static FNC exploits information from all dFNC states by aggregating features which together exhibit different properties than dynamic features. This can explain why the dynamic analysis replicated only two out of five effects previously found in the static analysis (Vergara et al., 2017), but with a higher effect size since the features might be specific to State 2. Note also that individual state connectivity is only one dimension of dFNC, for example, occupancy rate is a parameter that is not possible to estimate in static FNC but which represents a natural parameter to study when analyzing resting fMRI data. Fig. 2 also displays a matrix with all the *t*-values obtained indicating the FDR significant ones with circles. The *t*-value matrix portrays a trend of increased connectivity between cerebellum and cerebrum, but also an uncorrected trend including connectivity differences in DMN with SEN; LAN with SEN and VIS; ECN with SAL, VIS and SEN.

**Fig. 3:**
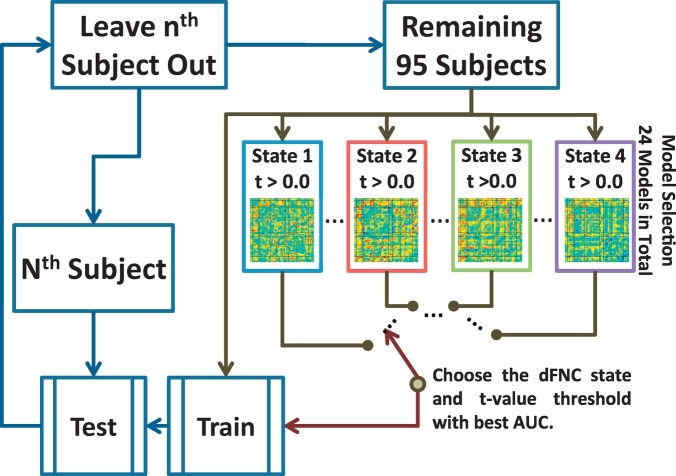
Schematic of the nested LOOCV loop used to identify the optimal state and feature selection threshold. Displayed matrices consist of *t*-values for each state. Features were selected using the *t*-values from two sample *t*-tests that were larger than one of the six thresholds [0.0 0.25 0.50 0.75 1.0 2.0]. In total there were 24 classification models (6 *t*-thresholds times 4 dFNC states). In this figure, the three dots between models indicate the existence of the other models.

We performed an initial classification on dFNC without feature selection separately for each state to get an idea of the predictive power for each state. Classification accuracy for each state was: State 1 (52%), State 2 (92%), State 3 (58%), and State 4 (52%). Because State 2 exhibited the largest AUC as well as significant group differences, we included the mean LSVM weights matrix in Fig. 2. Since one set of 1128 LSVM weights was obtained from training the classifier on each LOOCV loop, each weight was averaged over the 89 iteration outcomes. Each weight represents the importance of a feature in the classification performance. As expected, the two significant dFNC differences had also high LSVM weights. Another similarity is that dFNC and LSVM weights among RSNs in SEN and VIS groups are overall not significant and weak contributing little with the classification. Although State 2 exhibits the largest AUC and largest number of subjects (89), it is necessary to cross validate such selection. In addition, we would not be able to classify all 96 samples using only State 2. Similar to our previous report (Vergara et al., 2017), we applied a nested leave-one-out cross validation (LOOCV) loop (2 loops) to optimize the choice of the best state to classify the left out sample. We also added a feature selection step implemented using *t*-value thresholds. In this implementation, dFNC cells with absolute *t*-values (2 sample *t*-tests for group difference in the training set) larger than a threshold were selected. Six different [0.0 0.25 0.50 0.75 1.0 2.0] thresholds were tested. The inclusion of the 0.0 threshold allows comparing the selection of all features against selecting a fewer number of features. After leaving one sample out, twenty four different configurations (4 states and 6 *t*-thresholds) were tested using additional and nested LOOCV loops. The nested LOOCV is illustrated in Fig. 3.

**Table 2:** Significant group differences of dFNC in State 2. The two *t*-test results assed FDR correction. The table displays the original (uncorrected) *p*-values.

RSN	X	Y	Z	RSN	X	Y	Z	*t*-Value	*p*-Value
R Lob.VIIa Crus I	34	−77	−31	SMA/paracentral (BA6)	10	−26	66	5.04	2.5e-6
				Regression results	Sex	Age	Edu	WTAR	
				Betas	0.12	0.004	−0.04	0.0003	
				*p*-Values	0.07	0.26	0.03	0.94	
Lobule VI	10	−61	−25	SMA/paracentral (BA6)	10	−26	66	4.23	5.8e-5
				Regression results	Sex	Age	Edu	WTAR	
				Betas	0.19	0.003	−0.01	0.0006	
				*p*-Values	0.01	0.40	0.47	0.91

**Fig. 4:**
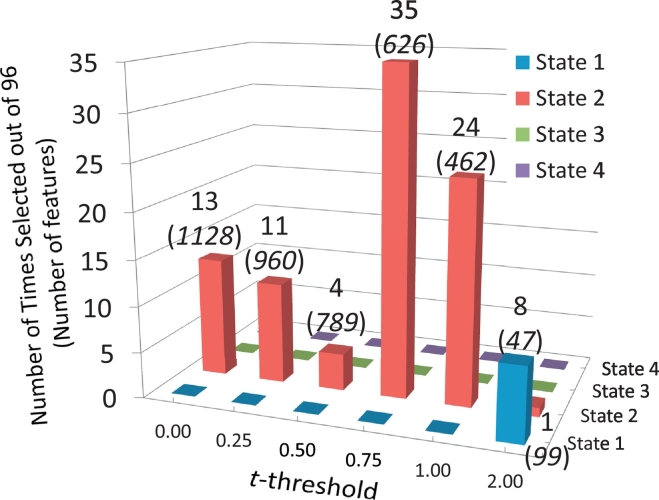
Histogram of classification performance (AUC) obtained using the nested loop of Fig. 3. State 3 and State 4 were never selected to classify the left out sample. State 2 was selected 88 times with the *t*-threshold of 0.75 as the preferred choice. State 1 was the second choice, which in most cases replaced missing subject data in State 2. The number of features is displayed in parenthesis below the number of times selected.

Fig. 4 displays a histogram of choices from the nested LOOCV in Fig. 3. Only State 1 and State 2 were chosen for classification. These two states have lower dFNC strength than State 3 and State 4. State 2 has the largest group differences, as detected by the significant *t*-tests, providing evidence to prefer this state for classification. State 1 followed State 2. However, significant differences were not detected in State 1, similar to what happened in State 3 and State 4. The total AUC considering all samples, states and thresholds was 87.5% with State 2 driving the performance as the preferred state. For comparison, we applied nested LOOCV and LSVM to the static FNC matrix. Nested LOOCV was used to select the appropriate *t*-threshold used in feature selection. Although this result has been previously reported in (Vergara et al., 2017) with an AUC of 84%, it was necessary to recalculate this value because the preprocessing was different for the current analysis. Specifically, despiking was performed by interpolation instead of censoring spiky time courses. In addition, the number of components is not the same. Nevertheless, the sFNC classification was 82% which is very similar to the value previously reported. We utilized ten thousand bootstrap iterations for both sFNC and dFNC and compared the results utilizing a *t*-test resulting in a significant performance difference. A histogram of the bootstrap outcome is presented in Supplementary Fig. 5.

## 4 Discussion

We have previously determined that FNC contains important information useful for identifying subjects with mTBI during the semi-acute stage (Vergara et al., 2017). However, the FNC in that study was static, i.e. calculated over a period of 5 min. Static connectivity demonstrated higher classification accuracy when compared to other modalities on the same cohort, but it does not consider temporal variations of connectivity. This static assumption ignores the dynamic properties of the brain. In contrast, the results presented in this current work include brain dynamics in an attempt to improve classification performance. The result was a significantly higher performance for the dFNC (87.5%) as compared to the sFNC (82.0%). The better performance achieved by dFNC was likely due to identification and separate analysis of each state. Numerical results indicated the existence of optimal dFNC states for classification. When the LSVM was applied within State 2, the performance was higher (92%) compared to classification utilizing all available states. Furthermore, State 2 not only was the sole state with significant group differences in connectivity, but was also consistently chosen to classify the testing samples when possible. These outcomes lead us to believe that State 1, State 3 and State 4 were not particularly useful for classification. The main characteristic of State 3 and State 4 was their low number of samples. Little can be done against this sample deficiency since the dFNC analysis is data driven having an unpredictable number of samples on each state. However, State 1 did not suffer from the curse of sample deficiency indicating a dFNC state with little information useful for classification. Arguably, the use of dFNC analysis might have worked as a way to separate useful features (in this case State 2 features) from less useful features (specifically those in State 1).

Classification features were selected based on correlation values requiring two RSNs to be defined thus hampering feature selection based on RSNs. Since the features were estimated from correlations, each requiring a pair of RSNs, there was no RSN that could be discarded without eliminating a chosen correlation (or selected feature) with another RSN. This is in line with the idea that functional connectivity effects in mTBI patients can be spread across many parts of the brain (Iraji et al., 2016; Stevens et al., 2012). On the other hand, our data suggests that not all connectivity pairs are necessarily important for classification. The most consistently selected *t*-threshold removed about half of the available features. The *t*-thresholds 0.75 and 1.00 were the preferred ones indicating that many dFNCs in State 2 might have added noise rather than predictive information. Not surprisingly, feature selection tended to select the states and cells with higher group differences. Furthermore, Fig. 2 illustrates the existing match between *t*-values and feature weights revealing similarities between the two patterns. Both measures point to connectivity values between sensorimotor and cerebellum areas as providing large group differences (*t*-values) and important classification features (large weights). Another common characteristic is that connectivity values between sensorimotor and visual RSNs provided weaker and fewer features than sensorimotor with cerebellum RSNs. In contrasts, many features in the executive control network scoring higher than the feature selection threshold (*t*-values > 1.00) exhibited relatively weak LSVM weights. The simplest way of explaining this difference is the lack of significant *t*-values across the ECN group also detected by the LSVM algorithm. Another view is that LSVM does not assume Gaussian distributed values. The particular objective of LSVM is to find a hyper-plane that separates HC from mTBI samples disregarding of its distribution (Cortes and Vapnik, 1995). This objective imprints differences from regular *t*-statistics including a natural way of coping with outliers thanks to LSVM's soft margin parameter (Ben-Hur et al., 2001). We note that State 1 was selected some few times with *t*-threshold larger than 2.00. The LSVM algorithm likely preferred State 1 over State 3 or State 4 because of a number of samples comparable with State 2.

As explained earlier, dFNC and its static counterpart may yield very different results. The separation of dynamic states, out of their integrated sFNC condition, involves non-linear processes such as the use of clustering algorithms that complicates a mathematical one-to-one mapping. Current dFNC outcomes do not include a significant difference between angular gyrus and precuneus as formerly found in static FNC (Vergara et al., 2017). Likely, the effect previously found was diluted and distributed among the dynamic states weakening its significance. The significant changes of dFNC strength in our analysis involved cerebellum and sensorimotor areas. In addition, there was a non-significant trend (after FDR correction) of increased connectivity between cerebellum and almost all cortical areas, as displayed in Fig. 2. In contrast to the angular-precuneus case, effects found in State 2 agree with the static FNC results confirming its importance in differentiating HC and mTBI samples. These high *t*-values associated to cerebellum, considering they are higher than the maximum feature selection threshold of 2.0, were critical for the classification performance obtained. Some studies have reported a similar increase in connectivity between these two areas for patients. Early studies report altered deactivations in the cerebellum and sensorimotor areas (Kasahara et al., 2010) consistent with our observations. Nathan et al. found increased connectivity between cerebellum and SMA (Nathan et al., 2015). Stevens et al. found correlation between the connectivity of the cerebellum and post concussive complaints (Stevens et al., 2012). Unfortunately, the cerebellum has not been included in several studies looking at functional connectivity. For example, Iraji et al. explored the whole brain without looking at the cerebellum (Iraji et al., 2016). A similar outcome was reported by Vakhtin et al. where many functional connectivity differences were found, but the cerebellum was omitted (Vakhtin et al., 2013). At this point, it seems that a closer look at the cerebellum in more recent studies reveals its effects in TBI patients (Palacios et al., 2017). Slobounov et al. reported an increased cerebellar activation in mTBI patients (Slobounov et al., 2010). This atypical cerebellar activation along with the increases in functional connectivity found between cerebellum and SMA (Nathan et al., 2015; Vergara et al., 2017) seems to coincide with abnormal anatomical connectivity in the cerebellar peduncle (Mac Donald et al., 2011; Sidaros et al., 2009). Observed functional changes are likely a reaction to anatomical insults in the white matter linking cerebellum with cortical areas. This should be evaluated in future work. However, results presented here indicate a dynamic effect that might explain to some degree differences in observed outcomes. First, significant group differences were only observed in State 2 between cerebellum and sensorimotor areas. Second, the number of subjects in State 2 was larger than in any other state. These two characteristics of the sample, only observed after dFNC analysis, can explain why the cerebellar-sensorimotor is predominant in dFNC, but also in a static FNC analysis previously reported (Vergara et al., 2017). We argue that if State 2 were detected in very few subjects then the final classification performance (87.5% AUC) would have not achieved the performance observed. Following our previous discussion, if State 2 were observed in all subjects then the performance would have reached a similar performance as that observed in State 2 alone (92% AUC). Our conclusion is that State 2 drives both the observed group differences and the classification performance.

One limitation of this work is the difficulty of measuring all dFNC states in a given subject. As displayed in Supplementary Fig. 3, some subjects spent most of the time in a single state (not necessary State 2) for the duration of the scan. Since State 2 drives the classification performance, the absence of this state in the fMRI scan can substantially limit its probability of correct mTBI detection. Whether dFNC abnormalities in State 2 might be a temporary disturbance remains an open question. Future work using longitudinal data should address this possibility. An important topic to be addressed in the future is to determine if State 2 is always present during the semi-acute state. In our cohort, five minutes collection time could have been too short to observe State 2 in all participants. If that is the case, a longer fMRI collection time, possibly 10 or 15 min, could help solve the issue. Another solution would be to collect data until dFNC State 2 is detected and measured. This would require the development of real time techniques useful in practical applications. Another possibility is to develop techniques that favor the detection of certain dynamic states. A first attempt is the use of mental training which increases dwelling in a dFNC state of focused attention (Gonzalez-Castillo and Bandettini, 2017; Mooneyham et al., 2017). This evidence suggests that future development could facilitate the application of dFNC state specific methods.

The following are the supplementary data related to this article.

Supplementary Fig. 1 Spatial maps for the selected resting state networks (RSNs). The figure includes MNI coordinates for each RSN. Supplementary Fig. 1

Supplementary Fig. 2 This figure displays the Cluster Validity Index resulting from running k-means with different number of clusters. Using the elbow criteria we selected four clusters. Supplementary Fig. 2

Supplementary Fig. 3 State dwelling per subject. Supplementary Fig. 3

Supplementary Fig. 4 Mean dFNC matrices for each group of HC and mTBI samples. The far right matrices are the *t*-value maps. Finding differences by visual inspection is difficult, but the *t*-value matrices help understanding the general difference patterns. Supplementary Fig. 4

Supplementary Fig. 5 Histograms of bootstrapped classification accuracies for static (sFNC) and dynamic (dFNC) functional network connectivity. Each bootstrap consisted of 10,000 iterations. A *t*-test (*t* = 101, df = 19,998, and sd = 3.62%) indicated a significant difference. Supplementary Fig. 5
