# Supplementary material for: Dynamic functional network connectivity discriminates mild traumatic brain injury through machine learning
Source: Neuroimage Clin. 2018 Mar 15;19:30–7. doi: 10.1016/j.nicl.2018.03.017 (PMC6051314; doi:10.1016/j.nicl.2018.03.017)

Supplementary Figure 1: Spatial maps for the selected resting state networks (RSNs). The figure includes MNI coordinates for each RSN.

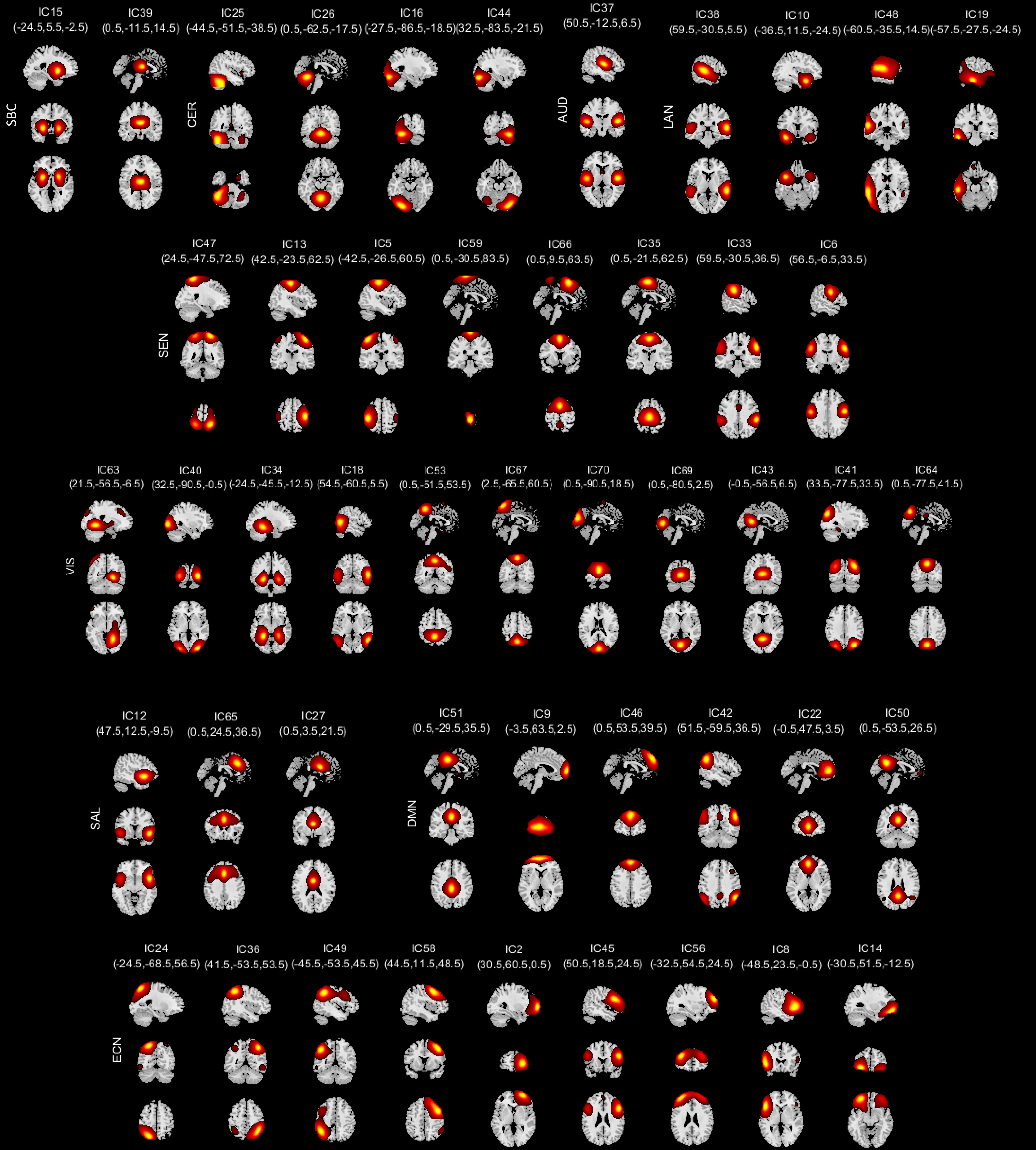

Supplement: Supplementary Fig. 1 — Spatial maps for the selected resting state networks (RSNs). The figure includes MNI coordinates for each RSN. [file mmc1.pdf]
