# Supplementary material for: Dynamic functional network connectivity discriminates mild traumatic brain injury through machine learning
Source: Neuroimage Clin. 2018 Mar 15;19:30–7. doi: 10.1016/j.nicl.2018.03.017 (PMC6051314; doi:10.1016/j.nicl.2018.03.017)
Supplement: Supplementary Fig. 2 — This figure displays the Cluster Validity Index resulting from running k-means with different number of clusters. Using the elbow criteria we selected four clusters. [file mmc2.pdf]

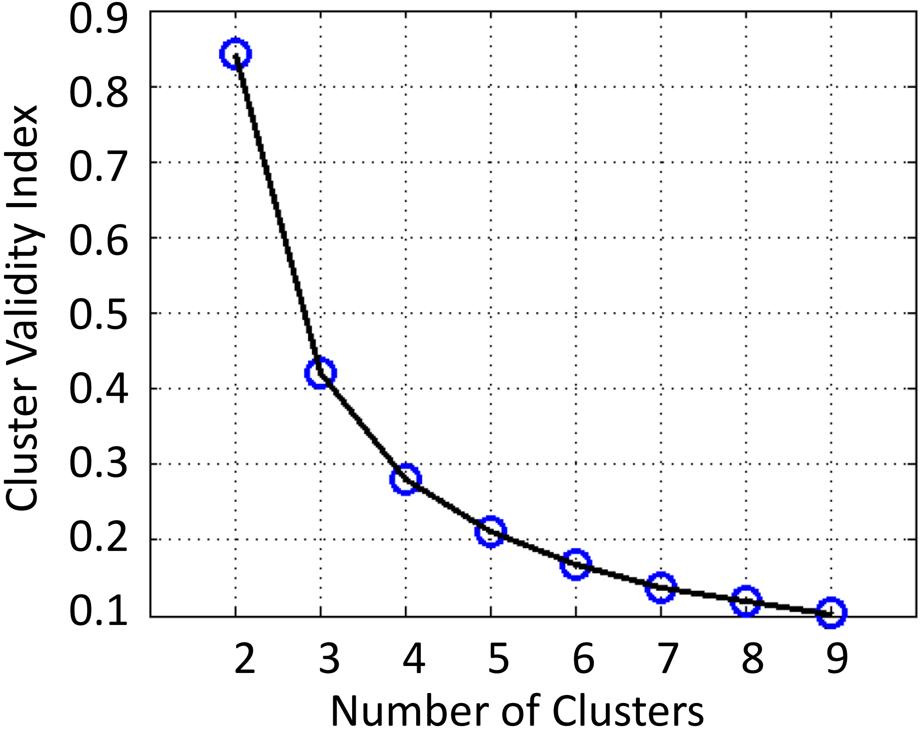

Supplementary Figure 2. This figure displays the Cluster Validity Index resulting from running k-means with different number of clusters. Using the elbow criteria we selected four clusters.
