# Supplementary figures and images for: Dynamic functional network connectivity discriminates mild traumatic brain injury through machine learning
Source: Neuroimage Clin. 2018 Mar 15;19:30–7. doi: 10.1016/j.nicl.2018.03.017 (PMC6051314; doi:10.1016/j.nicl.2018.03.017)

# Supplementary Figure 3: State Dwelling per Subject

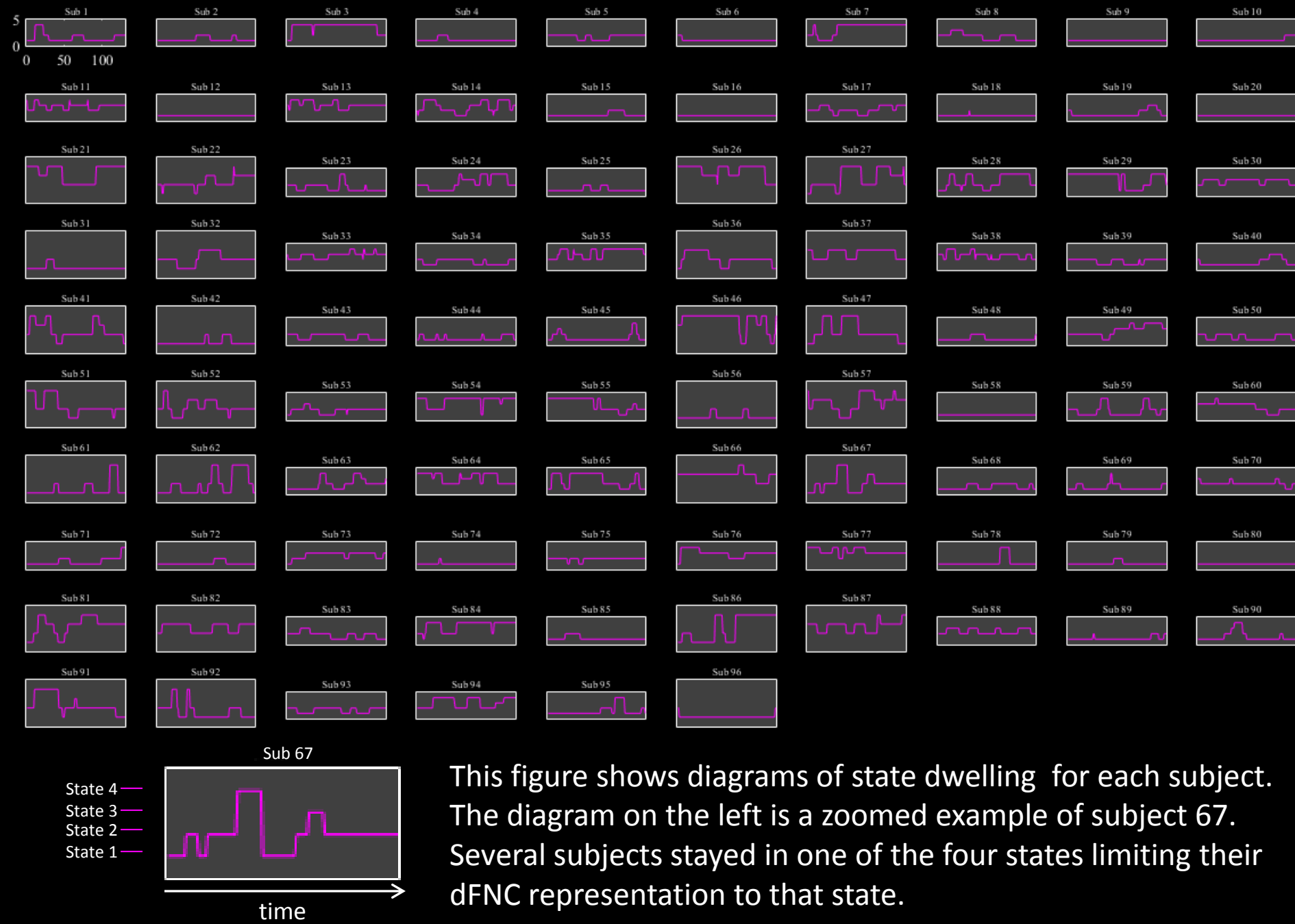

Supplement: Supplementary Fig. 3 — State dwelling per subject. [file mmc3.pdf]
