# Supplementary material for: Dynamic functional network connectivity discriminates mild traumatic brain injury through machine learning
Source: Neuroimage Clin. 2018 Mar 15;19:30–7. doi: 10.1016/j.nicl.2018.03.017 (PMC6051314; doi:10.1016/j.nicl.2018.03.017)

Supplementary Figure 4. Mean dFNC matrices for each group of HC and mTBI samples. The far right matrices are the *t*-value maps. Finding differences by visual inspection is difficult, but the *t*-value matrices help understanding the general difference patterns.

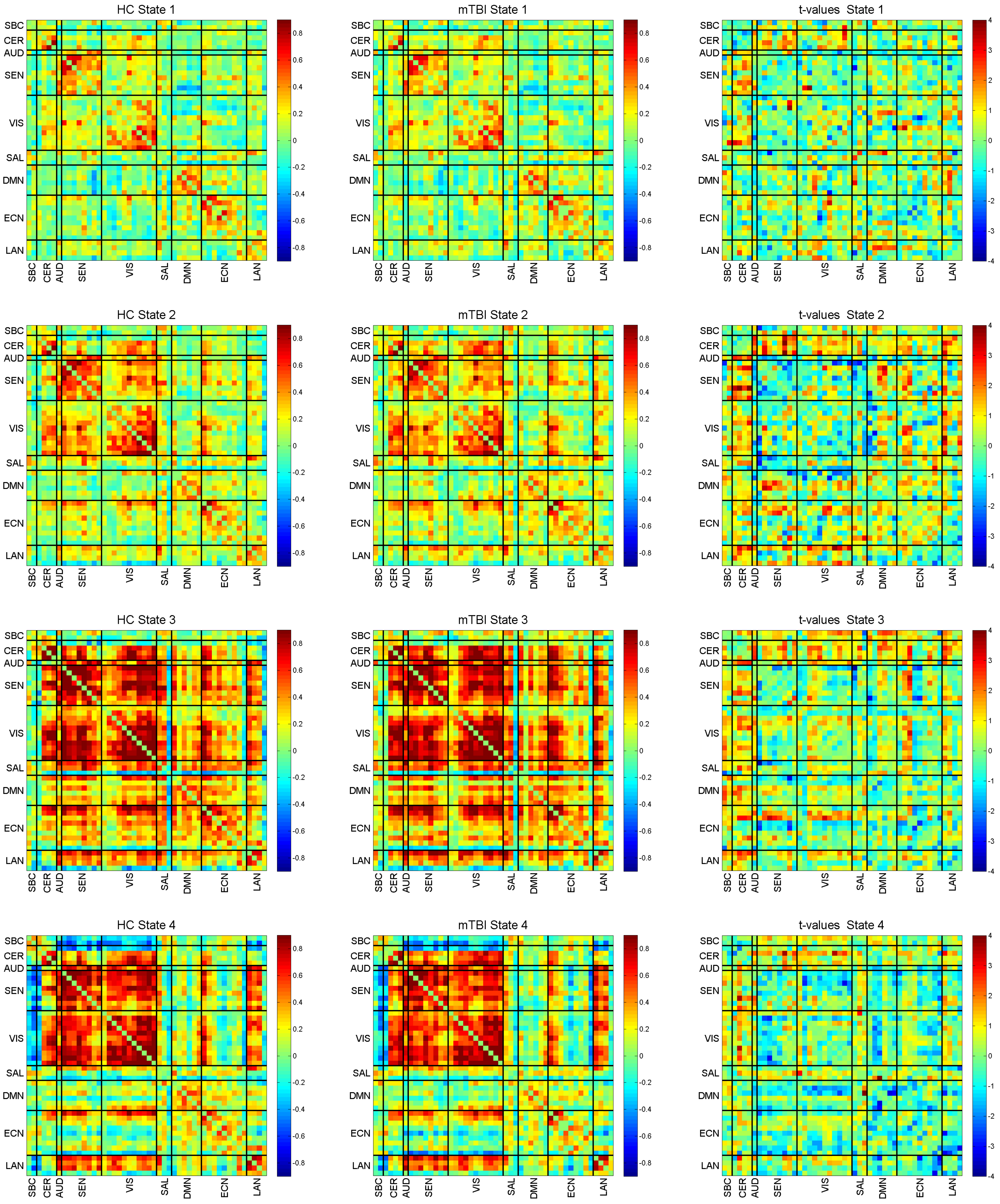

Supplement: Supplementary Fig. 4 — Mean dFNC matrices for each group of HC and mTBI samples. The far right matrices are the t-value maps. Finding differences by visual inspection is difficult, but the t-value matrices help understanding the general difference patterns. [file mmc4.pdf]
