# Supplementary material for: Dynamic functional network connectivity discriminates mild traumatic brain injury through machine learning
Source: Neuroimage Clin. 2018 Mar 15;19:30–7. doi: 10.1016/j.nicl.2018.03.017 (PMC6051314; doi:10.1016/j.nicl.2018.03.017)
Supplement: Supplementary Fig. 5 — Histograms of bootstrapped classification accuracies for static (sFNC) and dynamic (dFNC) functional network connectivity. Each bootstrap consisted of 10,000 iterations. A t-test (t = 101, df = 19,998, and sd = 3.62%) indicated a significant difference. [file mmc5.pdf]

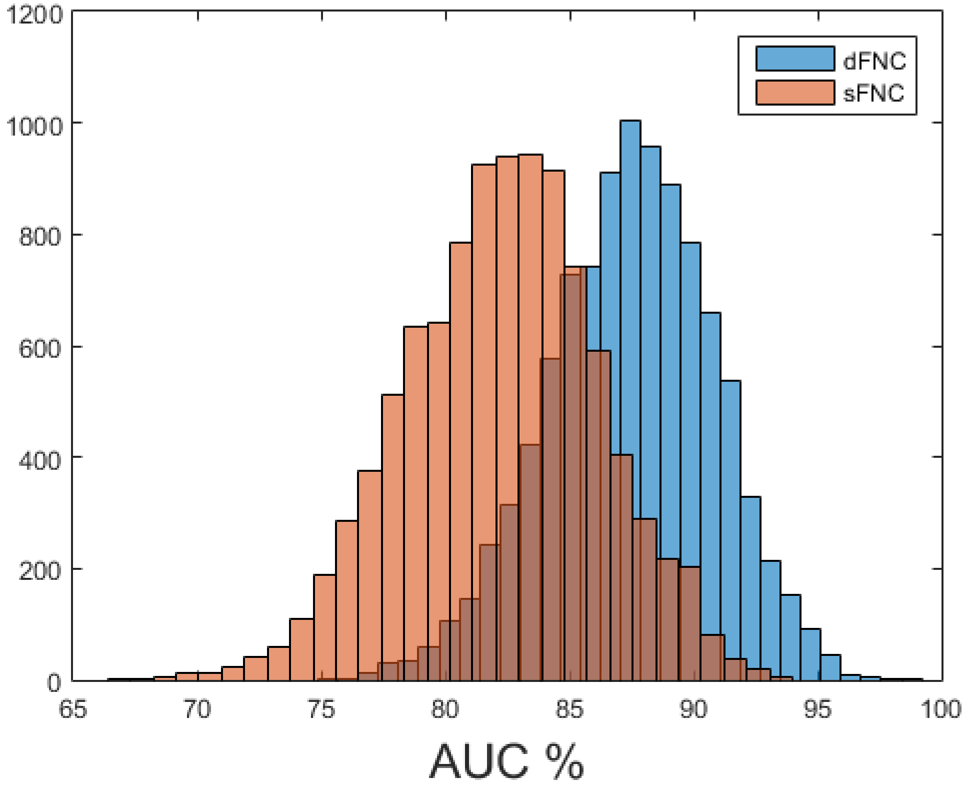

Supplementary Figure 5. Histograms of bootstrapped classification accuracies for static (sFNC) and dynamic (dFNC) functional network connectivity. Each bootstrap consisted of 10000 iterations. A t-test (t=101, df=19998, and sd=3.62 %) indicated a significant difference.
